# Induced pluripotent stem cell-derived cardiomyocytes—more show than substance?

**DOI:** 10.1007/s12551-023-01099-w

**Published:** 2023-07-19

**Authors:** Beth Ormrod, Elisabeth Ehler

**Affiliations:** 1 School of Cardiovascular and Metabolic Medicine and Sciences, King’s College London, London, SE1 1UL UK; 2 Randall Centre for Cell and Molecular Biophysics (School of Basic and Biosciences), Room 3.26A, New Hunt’s House, Guy’s Campus, London, SE1 1UL UK; 3 British Heart Foundation Centre of Research Excellence, King’s College London, London, SE1 1UL UK

**Keywords:** Human-induced pluripotent stem cells, Cardiomyocytes, Sarcomere, Engineered heart tissue, M-band

## Abstract

Cardiomyocytes that are derived from human-induced pluripotent stem cells (iPSC-CM) are an exciting tool to investigate cardiomyopathy disease mechanisms at the cellular level as well as to screen for potential side effects of novel drugs. However, currently their benefit is limited due to their fairly immature differentiation status under conventional culture conditions. This review is mainly aimed at researchers outside of the iPSC-CM field and will describe potential pitfalls and which features at the level of the myofibrils would be desired to make them a more representative model system. We will also discuss different strategies that may help to achieve these.

Human-induced pluripotent stem cells (iPSC) can be generated relatively easily using minimally invasive techniques such as skin biopsies or even cells isolated from urine (reviewed in (Karakikes et al. 2015). They have regained the ability to differentiate into cell types of all three germ layers (endoderm, ectoderm and mesoderm) and thus provide a potentially unlimited supply of differentiated cells. Numerous well-characterised iPSC lines exist (Kilpinen et al. 2017) and the cells can also be generated from patients of interest, meaning that the cellular phenotype of a particular disease can be studied with a genetic background that is matching the original patient. This makes them a potentially exciting tool to delineate disease signalling pathways at the cellular level. Also for the field of cardiovascular disease, iPSC technology has become a game changer since all the major cell types can be differentiated (cardiomyocytes, endothelial cells, smooth muscle cells) and their identity can be even driven further towards a specific cell type identity (arterial and venous-like endothelial cells; atrial or ventricular cardiomyocytes (Du et al. 2015; Rosa et al. 2019; Yang et al. 2016)).

One problem of iPSC-CM that are differentiated using commonly used techniques (Burridge et al. 2015) is that they are stuck at a relatively immature stage of differentiation with a morphology and marker expression that is very similar to a cardiomyocyte in the early embryonic heart. Organ development in the case of the heart has to face unique challenges: It is the first organ that has to function in the developing mammalian embryo, while allowing concomitant growth from its embryonic to its adult size. The way this conundrum is solved in the heart is that the embryonic cardiomyocytes are differentiated sufficiently to carry out the contractile work, but they are also still able to undergo cell division to allow for increase in heart size. For this process to happen, the contractile structures, the myofibrils, must be disassembled and then they are reassembled immediately following cytokinesis (Ahuja et al. 2004). This growth mechanism (hyperplasia) goes on until the first week after birth in the mouse (Li et al. 1996), by which time it is replaced by a growth mechanism of up to 40× increase in individual cell size (hypertrophy; (Laflamme and Murry 2011; Leu et al. 2001).

At the same time, the cardiomyocytes increase their status of maturation, they upregulate the expression of adult isoforms of contractile proteins, switch their metabolism from mainly glucose to fatty acid use and also change the organisation of their plasma membrane and intracellular membranous compartment to ensure the maximally efficient arrangement of calcium handling units in regularly arranged SR and T-tubules (Ehler 2016; Franzini-Armstrong et al. 2005). An isolated adult cardiomyocyte basically looks a little bit like a brick (classically termed rod-shaped), with cell-cell contacts restricted to the bipolar ends, while its longer sides only have cell-matrix contacts, since they are ensheathed in extracellular matrix in the tissue, similar to the insulation of an electrical cable. It is characterised by an extremely sophisticated cytoarchitecture, with a paracrystalline arrangement of its contractile elements, the myofibrils, which is matched by an extremely regular organisation of the energy-supplying mitochondria and of plasma membrane domains on the lateral cellular surface to T-tubules (Ehler 2016; Franzini-Armstrong et al. 2005). This phenotype ensures maximum contractile efficiency but goes hand in hand with an inability of the cardiomyocytes to undergo cell division and a loss of regenerative capacity (Porrello et al. 2011). In a recent perspective, Metzger has proposed a more time-resolved pathway for these processes that need completion in order to achieve the summit of differentiation for iPSC-CM and has suggested that sarcomere maturation is the last step of physiological maturation (Metzger 2022). He took the switch from the expression of the slow skeletal isoform of troponin-I to cardiac troponin-I as the ultimate step. In this review, we will expand from this and discuss, which other sarcomeric proteins and isoforms can be used as markers for maturation. We will also describe several approaches that can be used to upregulate their expression.

## The sarcomere

The sarcomere is the basic unit of a contractile myofibril and is delineated by two Z-discs. These are transverse struts that are mainly characterised by the expression of the cross-linking protein alpha-actinin, but that also play a more dynamic signalling role thanks to the association of a plethora of other proteins with them (Frank et al. 2006; Gautel and Djinovic-Carugo 2016; Wadmore et al. 2021). The thin filaments, which are composed of actin and the contraction-regulating proteins tropomyosin, troponin-I, troponin C and troponin T, insert perpendicularly into the Z-discs and run at a precisely defined length towards the middle of the sarcomere. They overlap in parallel with the thick filaments, which are bipolar bundles composed of myosin molecules (myosin heavy and light chains) and the interaction of the myosin heads with the actin filaments leads to shortening of the sarcomeres and myofibril contraction. Regulation at the thick filament level is mediated by myosin binding protein-C (MyBP-C), which is associated with a subset of the myosin heads in the C-zone of the thick filament (Kampourakis et al. 2014). For successful assembly of a sarcomere, there is a need for additional longitudinal and transverse cytoskeletal components (Gautel and Djinovic-Carugo 2016). The longitudinal link is provided by the elastic filaments, which are composed of titin, where one individual titin molecule has its N terminus anchored at the Z-disc and then stretches all the way to the middle of the sarcomere. There the C-terminus of titin integrates the myosin tails together with another cytoskeletal protein, myomesin, in the M-band, a transverse strut that demarcates the middle of the sarcomere (Lange et al. 2020).

## Isoforms of contractile proteins: actin and myosin

**Fig. 1:**
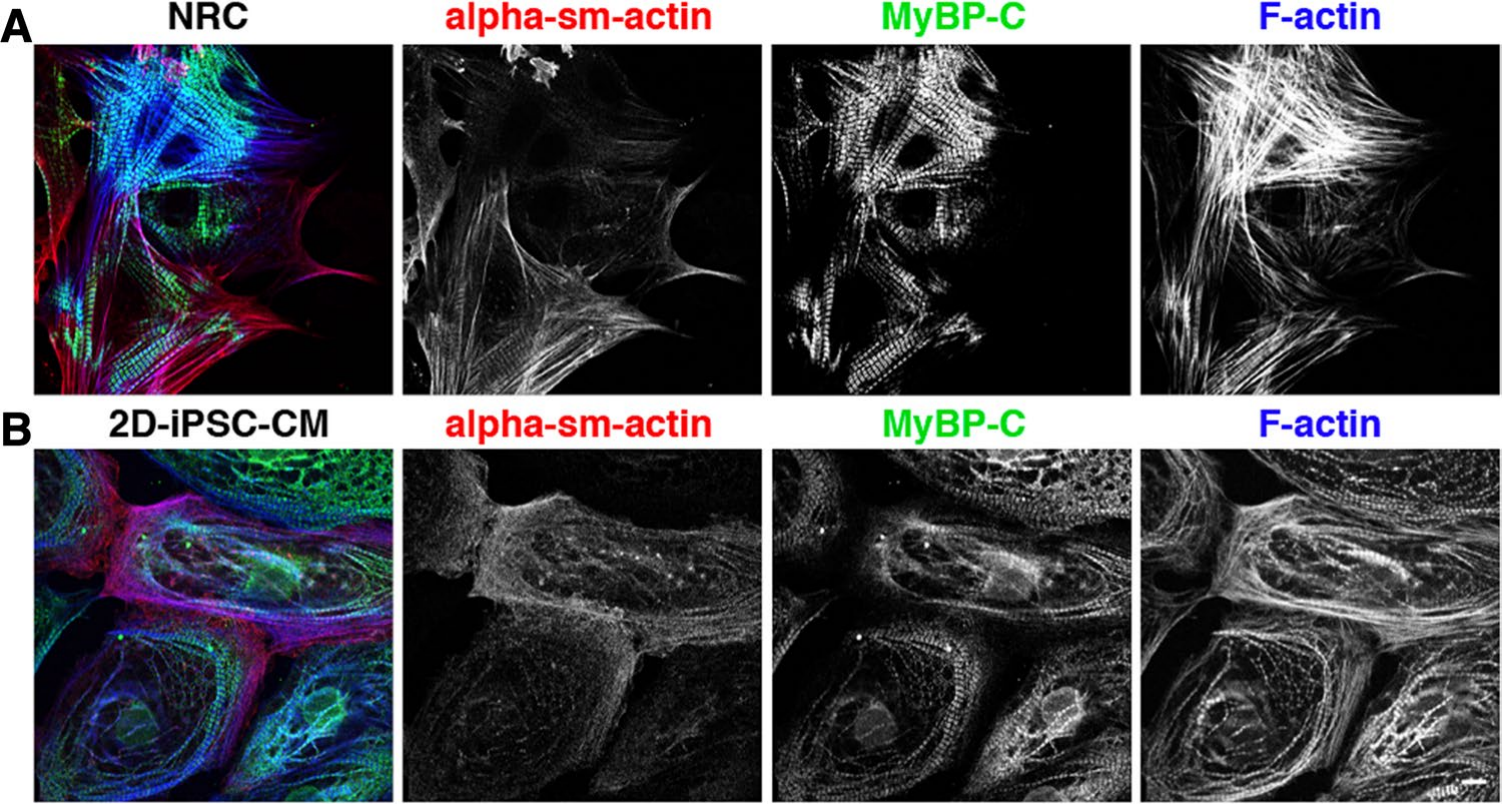
The morphology of iPSC-CM resembles embryonic cardiomyocytes. Confocal micrographs of primary cultures of neonatal rat cardiomyocytes (NRC; top row) and iPSC-derived cardiomyocytes (iPSC-CM; bottom row); immunostained for alpha-smooth muscle actin (red in overlay); MyBP-C (green in overlay) and using phalloidin to visualise F-actin (blue in overlay). The myofibrils of NRC are bulkier and stretch throughout the cytoplasm, while the myofibrils of the iPSC-CM are weedier and mainly found in the periphery of the cell. Scale bar is 10 μm

An integral element of myofibril maturation is isoform switching of sarcomeric proteins from foetal to adult isoforms via alternative splicing or transcriptional changes. In normal myocardium, three alpha-muscle actin isoforms are co-expressed, alpha-smooth muscle actin, alpha-skeletal muscle actin and alpha-cardiac muscle actin, each playing different functional roles. The amounts of alpha-muscle actin transcripts can vary depending on the developmental stage of cells, ageing and disease. In vivo, cardiomyocyte differentiation begins with transient expression of alpha-smooth muscle actin (Ehler et al. 2004; Ruzicka and Schwartz 1988). During development, alpha-skeletal muscle actin and alpha-cardiac muscle actin are co-expressed, until finally in the adult heart, alpha-cardiac muscle actin becomes the predominant actin isoform (Vandekerckhove et al. 1986), with alpha-smooth muscle actin exclusively expressed in smooth muscle cells of the vasculature. However, in the myocardium of patients with pathological cardiac hypertrophy, alpha-smooth muscle actin can be found (Black et al. 1991). When we compare iPSC-derived cardiomyocytes with the gold standard for cardiomyocytes in culture, primary cultures of neonatal rat cardiomyocytes (NRCs) using MyBP-C (Offer et al. 1973) as a myofibril marker, the myofibrils look much more differentiated in NRCs (Fig. 1). They stretch throughout the middle of the cell in more substantial bundles (see MyBP-C signal in Fig. 1A), while in iPSC-CMs they tend to be found as weedy, less well aligned myofibrils in the cellular periphery (Fig. 1B). The signal for alpha-smooth muscle actin is variable between different NRC, in some there are only minor amounts in the cellular periphery, potentially representing a culture artefact that is due to the need to attach to the culture dish, while in others, alpha-smooth muscle actin striations can clearly be detected also within more mature looking sarcomeres. In iPSC-CMs, most of the cells clearly express high levels of alpha-smooth muscle actin, which overlaps with the myofibrils at the cellular periphery. Cardiac troponins represent good markers of maturation as they switch from the foetal slow skeletal troponin-I to adult cardiac troponin-I (Bedada et al. 2014). iPSC-CMs have been reported to have patchy expression of cardiac troponin-I, contributing to their immature characterisation. During heart development, isoform switches are not only seen for classical constituents of the thin filaments as described above, but were recently also shown for actin-assembly regulating proteins such as members of the formin family. For example, the formin FHOD3 is expressed as a muscle-specific isoform that contains a longer insertion encoded by a single exon in striated muscle in the adult. However, the expression of this isoform is reduced in cardiomyopathy and only gets switched on late during development (Iskratsch et al. 2010; Iskratsch et al. 2013), making muscle FHOD3 also a potentially interesting maturity marker for iPSC-CM.

During human heart development, the cardiomyocytes initially express alpha-myosin heavy chain (encoded by MYH6) and only upregulate the expression of the mature isoform, beta-myosin heavy chain (encoded by MYH7) by 5 weeks of gestation (Reiser et al. 2001). This co-expression of embryonic and adult myosins is still seen when iPSC-CMs are analysed, as shown by Zuppinger et al. (2017). When grown in a more physiological 3D arrangement for example in cardioids, the cardiomyocytes still retain high levels of alpha-myosin heavy chain expression, indicating a lack of maturity at the level of the myofibril (Hofbauer et al. 2021; Selewa et al. 2020). Developing human heart myosin light chain 2 atrial (MLC2a; encoded by the MYL7 gene) is detected in all chambers, whereas postnatal heart myosin light chain 2 ventricular (MLC2v; encoded by MYL2) is confined to the ventricles. This chamber specificity continues into adulthood (Kubalak et al. 1994); therefore, MLC2v is considered to be a marker of cardiac myocyte maturity. iPSC-CMs have been reported to express both MLC2 isoforms, some express MLC2a alone, some MLC2v alone or a combination of both (Bedada et al. 2014).

## Transverse struts: Z-disc and M-bands

**Fig. 2:**
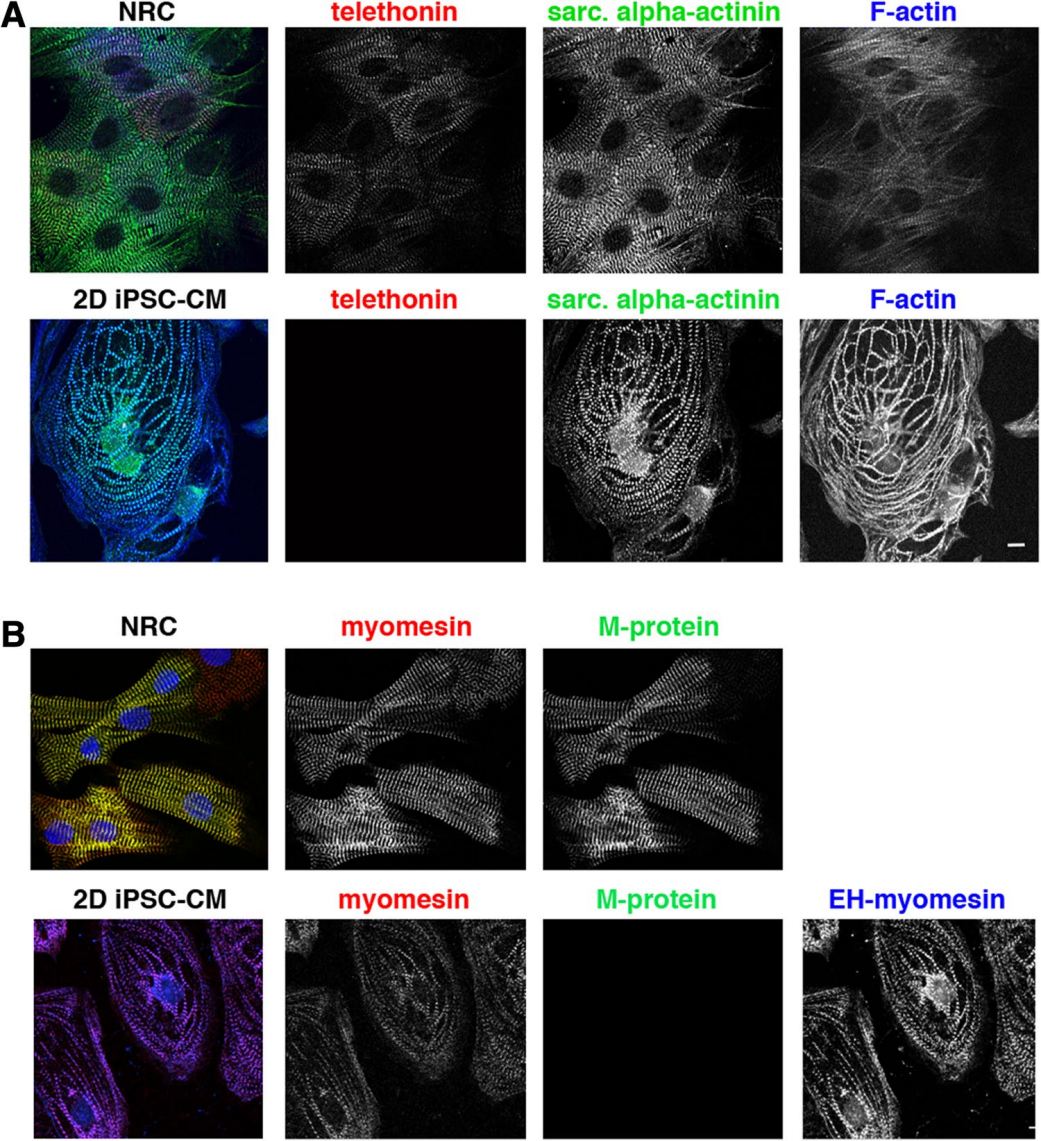
Telethonin and M-protein are useful markers for Z-disc and M-band maturity, respectively. Confocal micrographs of primary cultures of neonatal rat cardiomyocytes (NRC; top) and iPSC-derived cardiomyocytes (iPSC-CM; bottom); immunostained for telethonin (red in overlay in **A**) and M-protein (green in overlay in **B**). All myofibrils were visualised either by staining for the Z-disc protein alpha-actinin (green in **A**; F-actin, as visualised by phalloidin is blue) or by staining for the M-band protein myomesin (red in B; the embryonic heart specific splice variant EH-myomesin is shown in blue). The antibody against the EH-myomesin splice variant is specific for humans; therefore, no signal was obtained for the NRC. Scale bar is 10 μm

The best markers that we have identified for a mature status of the sarcomere are however found at the transverse linking structures, the Z-disc and the M-band. Telethonin, also known as titin-cap or T-cap, is a 19-kDa striated-muscle-specific Z-disc protein (Valle et al. 1997), that plays a major role in sarcomere assembly and stretch sensing (Mues et al. 1998). Telethonin has a unique beta-sheet structure which assembles in a palindromic fashion with the N-termini of two adjacent titin molecules (Zou et al. 2006). This titin-telethonin interaction has been demonstrated as the strongest protein-protein interaction to date (Bertz et al. 2009). Importantly, the incorporation of telethonin at the Z-disc via binding to titin is one of the final stages in maturation of the Z-disc (Wang et al. 2005), which may explain the lack of telethonin in iPSC-CM (Fig. 2A bottom). Even in NRCs only a subset of cells is positive for telethonin (red signal in Fig. 2A top).

**Fig. 3:**
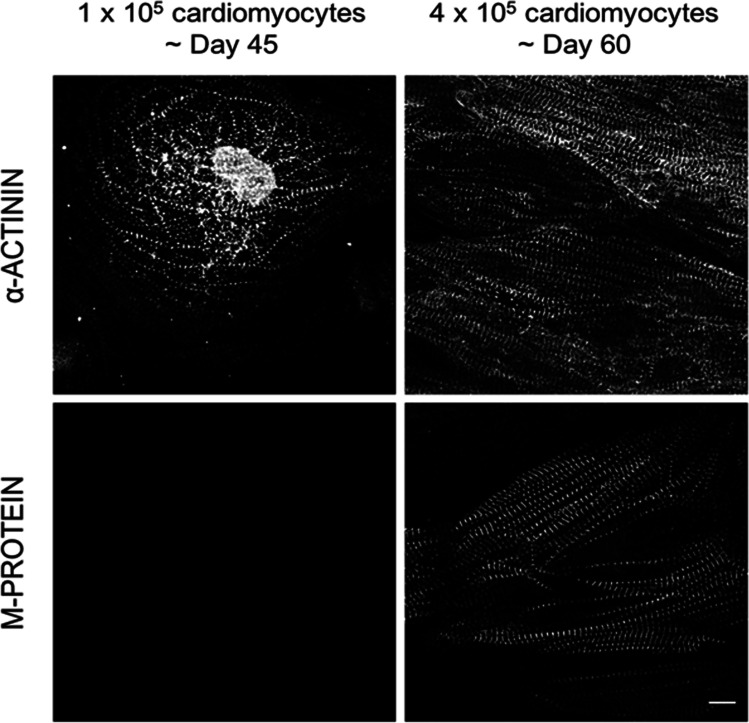
Prolonged and denser culture leads to the upregulation of M-protein expression in iPSC-CM. Confocal micrographs of iPSC-derived cardiomyocytes (iPSC-CM) grown for 45 or 60 days (left- and right-hand column, respectively) and immunostained for sarcomeric alpha-actinin (top row) and M-protein (bottom row). The myofibrils in the longer-term cultures are more mature and also display a signal for M-protein. Scale bar is 10 μm

The M-band is positioned at the centre of each sarcomere and links the titin with the thick (myosin) filaments. It is mainly composed of different members of the myomesin family, which are present in slightly different amounts, depending on the developmental stage of the muscle and the type. All sarcomeres will contain myomesin (encoded by MYOM1), but the expression of its more elastic splice variant EH-myomesin is only seen in embryonic heart and in slow twitch skeletal muscle (Agarkova et al. 2000; Agarkova et al. 2004), while the expression of M-protein (encoded by MYOM2) is specific for adult heart and fast twitch skeletal muscle (reviewed in (Lange et al. 2020). In NRC, the extent of cells that are positive for M-protein is slightly higher than those that are positive for telethonin (compare Fig. 2A with Fig. 3A), but there are still cells that are only positive for myomesin in their M-bands. For iPSC-CM in conventional short term 2D cultures, we detect no signal for M-protein and still a 100% expression of the embryonic splice variant of EH-myomesin with an antibody that is specific for human EH-myomesin.

**Fig. 4:**
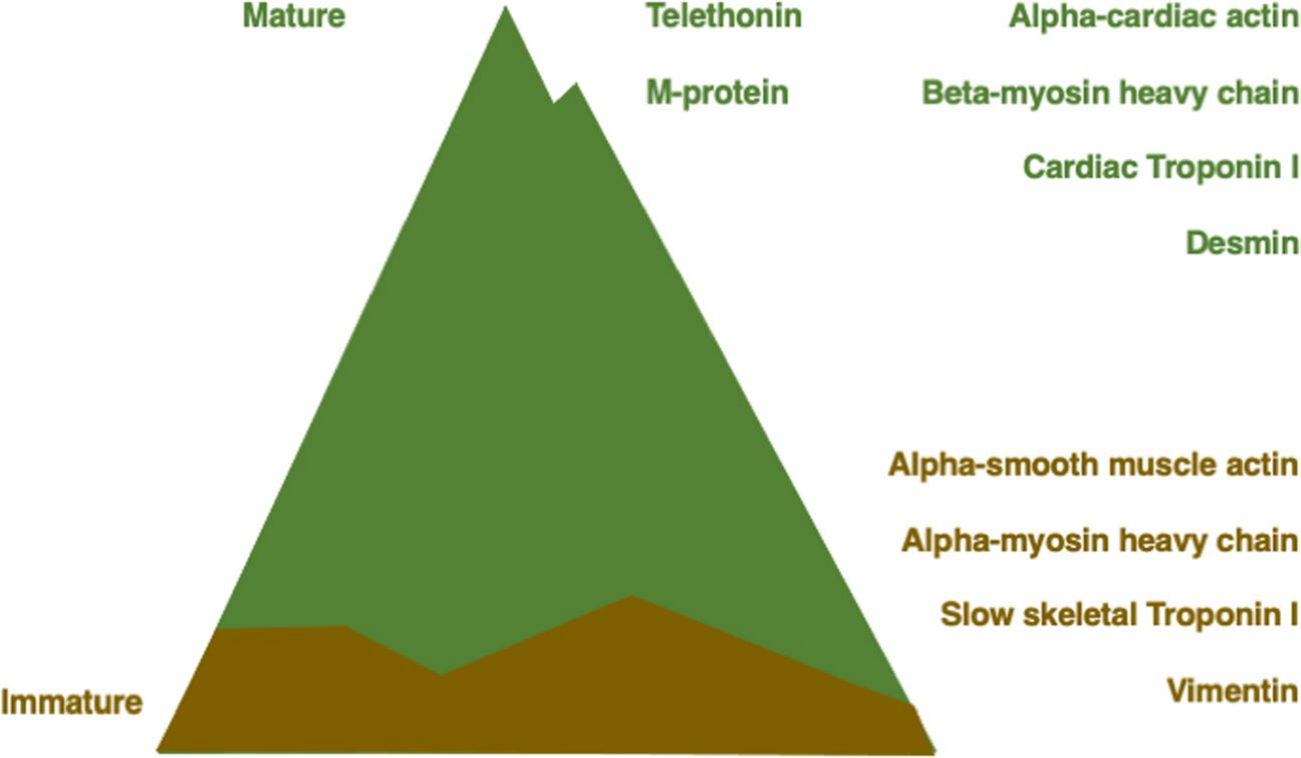
From immature swamps to the peak of maturity. Schematic drawing comparing selected sarcomeric markers for immature iPSC-CM at the bottom (brown) and for mature iPSC-CM at the top (green)

These observations suggest that the presence of telethonin at the Z-disc and of M-protein at the M-band are extremely useful indications for the maturity of an iPSC-CM and should be the targets to look out for (see Fig. 4).

## Forcing maturation

Which tricks can be used to force iPSC-CM to reach the summit of differentiation as defined by Metzger and expanded on above (Metzger 2022)? There are basically two approaches, that are commonly used, one is time and the other is environmental stimuli, by using growth conditions (growth factors; metabolic maturation; oxygen levels; extracellular matrix; other cell types) that are more similar to the environment the cells would be exposed to in the developing heart (for recent reviews, see Guo and Pu 2020; Karbassi et al. 2020).

One of the initial maturation studies simply increased the culture time of iPSC-CM in vitro (Lundy et al. 2013). Comparing early-stage iPSC-CMs, cultured for between 20 and 40 days, with late-stage iPSC-CMs, cultured between 80 and 120 days, dramatic differences with regard to maturation were observed in the late-stage iPSC-CMs. The cells were larger, more elongated, there was greater myofibrillar alignment and density, sarcomeres were more easily distinguished, and there was an increase in multinucleated cardiomyocytes. Functionally, better contractile performance and calcium handling was reported and key cardiac structural markers including connexin-43 and beta-myosin heavy chain were upregulated (Lundy et al. 2013). Taken together, these findings suggested an increased level of maturation could be achieved through simply allowing iPSC-CMs longer time in vitro culture. While prolonged culture is clearly effective, as also shown by Kamakura and colleagues who, after a year, detected a mature appearance of the M-band in electron micrographs (which is only achieved by the incorporation of M-protein; (Kamakura et al. 2013), this is not a time frame that is useful and financially viable for most studies.

The other main approach is to play around with environmental stimuli to create a surrounding for the iPSC-CM that favours a more mature phenotype. For example, in our hands, the combination of more dense plating and longer culture times (60 days) resulted in the upregulation of M-protein expression (as seen in Fig. 3). Other researchers have seen somewhat improved sarcomeric alignment in iPSC-CM that were cultured on nanogrids with a biomimetic interface (Carson et al. 2016). Faster maturation of iPSC-CM could also be induced by growing them on a cardiac mimetic matrix, which resulted in an increased expression of sarcomeric markers such as cardiac troponin-I and MLC2v, accompanied by increased numbers of mitochondria and a more mature metabolic and electrophysiological phenotype (Afzal et al. 2022).

In addition, 3D cellular aggregates have previously been shown to mature iPSC-CMs. They displayed downregulation of genes involved in glycolysis compared with 2D iPSC-CMs, as well as increased expression of genes involved in oxidative phosphorylation. Across multiple iPSC-CM lines, metabolic maturation was significantly increased, when compared with their 2D counterparts (Correia et al. 2018). Engineered heart tissues (EHTs) are an alternative 3D culture approach widely used within the cardiovascular field. They contain a scaffold matrix, which in most cases is a hydrogel populated by cells between attachment sites. EHTs were first described in 1997 with the use of embryonic chicken cardiomyocytes cultured inside collagen I or Matrigel hydrogels, as a way to measure contractile force (Eschenhagen et al. 1997). Several different EHT formats have been proposed, including positioning between two posts (Eschenhagen et al. 1997), positioning in a ring shape (Zimmermann et al. 2002) or positioning in cylinder constructs (de Lange et al. 2011). In addition, co-cultures of different cell types were shown to improve cardiac function in EHTs, as initial populations of highly purified cardiomyocytes resulted in cardiomyocyte loss within the EHT (Kensah et al. 2013). Several studies have tested the addition of endothelial cells and fibroblasts (Kensah et al. 2013; Stevens et al. 2009); however, using an unselected or mildly selected population of differentiated iPSCs has also been reported. Post-differentiation, just 30–40% of cells are cardiomyocytes, suggesting a yield of a mixed population of cells. In one particular study, the other cell types were identified as being predominantly fibroblasts and a role for the fibroblasts in successful formation of the EHTs was postulated, likely due to their production of extracellular matrix and secretion of growth factors (Schaaf et al. 2011).

One key feature notably absent from iPSC-CMs relative to adult cardiomyocytes are T-tubules. The T-tubule system is a complex network of interconnected tubules and membranes that are contiguous with the extracellular space (Franzini-Armstrong and Porter 1964). They are rich in ion channels and the invaginations allow for rapid excitation-contraction coupling, which is necessary for efficient relay of the action potential in large cells such as ventricular cardiomyocytes (Soeller and Cannell 1999). One relatively recent study identified that a combination of matrix components and hormones resulted in iPSC-CMs displaying some T-tubules (Parikh et al. 2017), while functionally they still lacked the abundance and detailed organisation found in adult ventricular cardiomyocytes and also exhibited slower Ca^2+^ cycling. Another approach grew iPSC-CM in rectangular-shaped 3D scaffolds and demonstrated improved myofibril alignment and sarcolemma remodelling, resulting in better calcium handling and enhanced spontaneous beating activity (Silbernagel et al. 2020). Combining iPSC-CMs, cardiac fibroblasts and cardiac endothelial cells into scaffold-free 3D microtissues has been shown to enhance cardiomyocyte maturation (Giacomelli et al. 2020). The addition of cardiac fibroblasts specifically, to iPSC-CMs in 3D microtissues, improved sarcomeric structures. T-tubules could be observed, contractility was enhanced, there was increased coupling of iPSC-CMs and cardiac fibroblasts through connexin-43 gap junctions and they were electrophysiologically mature (Giacomelli et al. 2020).

Making the cells “work” by applying forces to induce mechanical stretch or by using electrical stimulation is another way of triggering maturation (for review, see Swiatlowska and Iskratsch 2021). Mechanical stretch on its own or combined with electrical stimulation improved the expression ratio of mature isoforms of thick filament proteins such as beta-myosin heavy chain and ML2v as well as sarcomere alignment (Kreutzer et al. 2020; LaBarge et al. 2019). When iPSC-CMs in EHTs were paced with biphasic pulses and compared with non-stimulated EHTs, the stimulated EHTs had a higher cardiomyocyte density, increased connexin-43 expression and improved sarcomere ultrastructure, including the presence of M-bands. The functional maturation was also evident by decreased spontaneous beat activity and increased force responses to isoprenaline (Hirt et al. 2014). Another study explored the role of biomimetic mechanical and electrical stimulation on EHTs. EHTs were subjected to electrical field stimulation while on flexible poles to facilitate auxotonic contractions. The EHTs had improved calcium storage and release capacity of the sarcoplasmic reticulum, with enhanced T-tubule formation, suggesting electro-mechanical stimulation at a physiological frequency encouraged functional maturation of cardiomyocytes (Godier-Furnemont et al. 2015). A physical conditioning approach, which involved increasing intensities over 2 weeks, followed by a further week at 2-Hz stimulation, achieved iPSC-CMs in EHTs with a gene expression profile closely related to adult cardiomyocytes and a well-developed ultrastructure, including T-tubules (Ronaldson-Bouchard et al. 2018).

Changing the energy metabolism or the levels of oxygen the cells are exposed to can also be employed to stimulate maturation. For example, exposing the cells to a mixture of fatty acids helped to improve maturity (Correia et al. 2017; Yang et al. 2019). Conventional culture media contain high levels of glucose, which activates hypoxia-inducible factor 1 alpha (HIF1alpha). Since downregulation of HIF1alpha activity after birth due to increased oxygen levels compared to the hypoxic environment in the uterus is an important step in cardiomyocyte maturation (Krishnan et al. 2008), this is counterproductive and inhibition of HIF1alpha was shown to promote iPSC-CM maturation, from a metabolic perspective as well as by increasing sarcomere organisation and contractility (Hu et al. 2018).

Activating signalling pathways that are switched on perinatally is another way to trigger maturation. When iPSC-CM are exposed to increased levels of the thyroid hormone T3 (triiodothyronine), myosin isoform switching, hypertrophic growth and improved calcium handling were seen (Yang et al. 2014). T3 together with glucocorticoid hormones was demonstrated to improve calcium kinetics accompanied by the presence of discernible T-tubules (Parikh et al. 2017). A very recent study has tried to harness signalling pathways that would occur during endogenous heart development more faithfully, which has resulted in iPSC-CM that even expressed telethonin and possess T-tubules (Dark et al. 2023).

In conclusion, a lot of iPSC-CM studies are hampered by a lack of maturity of the analysed cardiomyocytes and the high degree of heterogeneity between iPSC lines; differentiation potential even within the same line and protocol and distinct cardiomyocyte survival rates within 3D aggregates, which are well recognised by researchers within the field, but probably less obvious to researchers, who are less familiar with the potential pitfalls. Therefore, what the field urgently needs, is a careful evaluation of the extent of maturity of the cardiomyocytes that are analysed, so it becomes more straightforward to compare results between groups and approaches. As outlined in this review, the presence of sarcomeric alpha-actinin is just not good enough and a consistent analysis of the isoform expression of slow skeletal troponin-I versus cardiac troponin-I, alpha-myosin heavy chain versus beta-myosin heavy chain, MLC2a versus MLC2v, ideally supplemented with the investigation of the presence of the Z-disc and M-band maturity markers telethonin and M-protein is required to correctly place the iPSC-CM that are analysed in a particular experiment on the mountain path to maturity (Fig. 4). We conclude that there does not seem to be a single magical bullet to achieve adult-like maturity of iPSC-CM and that a combinatorial approach of 3D culture in the presence of physiological levels of oxygen and concomitant activation of perinatal maturation signalling pathways by growth factors and metabolic substrates is clearly the way to go to push iPSC-CM up the steep slope to the summit of mature myofibrils.
